# Christmas, acute ischemic stroke and stroke‐related mortality in Hungary

**DOI:** 10.1002/brb3.2104

**Published:** 2021-03-09

**Authors:** András Folyovich, Réka Mátis, Nadim Al‐Muhanna, Tamás Jarecsny, Eszter Dudás, Dorottya Jánoska, Mihály Pálosi, Anna K. Béres‐Molnár, Gergely Toldi

**Affiliations:** ^1^ Department of Neurology and Stroke Szent János Hospital Budapest Hungary; ^2^ Faculty of Public Governance and International Studies University of Public Service Budapest Hungary; ^3^ National Health Insurance Fund Budapest Hungary; ^4^ Department of Laboratory Medicine Semmelweis University Budapest Hungary

**Keywords:** anxiety, death shift, emotional impact, stress, thrombolysis

## Abstract

**Objectives:**

Risk factors for stroke include psychological effects, such as depression. Festive occasions (such as Christmas in Hungary) may carry a significant emotional impact and may therefore contribute to increased cardiovascular risk. Thrombolytic treatment of acute ischemic stroke has a narrow time window and allows for the precise assessment of stroke incidence.

**Materials & Methods:**

We analyzed anonymized national data of the number of thrombolytic treatments for acute ischemic stroke and the number of stroke‐related deaths between 1 January 2007 and 31 December 2016 in Hungary within 2‐day, 5‐day, and 1‐month periods preceding and following 24 December each year. Analysis of subgroups based on age (below and over 65 years) and sex was also performed.

**Results:**

The number of thrombolytic treatments was higher in all three periods preceding Christmas compared to the corresponding period that follows the feast. This increase was particularly prominent in men below 65 years of age. While overall stroke‐associated mortality was increased 1 month after Christmas, the death rate was higher a month before rather than after Christmas in men below 65 years of age and in women both below and over 65 years of age 5 days before Christmas.

**Conclusions:**

These findings may predominantly relate to emotional and psychological factors. In case of women, the anxiety secondary to festive preparations, while in men below 65 years, the increased psychological stress of providing financial security for the celebration may play an important role.

## Graphical abstract

The number of thrombolytic treatments for acute ischemic stroke is significantly higher in the 2‐day, 5‐day, and 1‐month periods preceding Christmas in Hungary compared to the corresponding period that follows the feast. This increase is particularly prominent in men below 65 years of age.

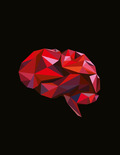

## 1 INTRODUCTION

Linking the onset of stroke with different environmental factors or events provides an opportunity for the recognition of pathogenic associations. The accumulation of cardiovascular events in winter is a well‐known phenomenon and can be well explained by pathophysiological causes (Azevedo et al., 1995; Tsementzis et al., 1991). The first workday of the week and the last workday of the month are vascular risk factors, which cannot be explained purely by biological impacts, but the role of psychological and sociological causes can also be suggested (Folyovich et al., 2016, 2018). The role of complex sociocultural factors can be identified as contributors to the increased risk of stroke among people with lower income (Reshetnyak et al., 2020). Based on this, it can be suggested that social events and feasts associated with significant emotional impact can influence the cardiovascular events. Literature data suggest that religious events postpone the death of patients in an otherwise serious condition to a date that is after the feast (Phillips & King, 1988; Phillips & Smith, 1990). The role of a particular social or religious festivity varies in different societies, and the presence and extent of the “death shift” phenomenon can reflect the spiritual attitude of a country. In Hungary, Christmas (typically Christmas Eve, 24 December) appears to be a feast that has the largest influence on the whole society. Although the Christian churches (in particular the Catholic church) predominate, Christmas in Hungary is traditionally also celebrated by atheists and people of other religions. In the present study, we therefore examined the influence of Christmas on the incidence of acute ischemic stroke and stroke‐related mortality.

## 2 METHODS

The precise determination of the onset of acute ischemic stroke is enabled by the analysis of the number of thrombolytic interventions performed, due to the narrow therapeutic window and rigorous administrative requirements. These two criteria counterbalance the disadvantage posed by the fact that only a relatively small rate of patients undergo such therapy. If a country's rate of thrombolytic interventions performed against the total number of acute stroke events is known, the absolute number of thrombolysis performed in a given period can well represent total stroke incidence. We analyzed anonymized data between 1 January 2007 and 31 December 2016 provided by the National Health Insurance Fund of Hungary and its predecessor in law. The study was exempt from institutional ethics approval as an anonymized national dataset was analyzed. The number of thrombolytic interventions was analyzed within 2‐day, 5‐day, and 1‐month periods preceding and following 24 December each year. In case of all three time periods, comparisons were conducted first by using the complete sample, which was followed by the analysis of subgroups based on age (below and over 65 years of age—actively employed and pensioners) and sex (males and females). For the periods before 24 December, comparative analysis between the above subgroups based on age and sex was also performed.

Data of the Hungarian Statistical Office were used to determine the stroke‐related mortality (International Classification of Diseases [ICD] I60‐67). During statistical analysis, first the whole sample was used in the case of all three time periods, which was followed by the analysis of subsamples based on age and sex as described above.

Event rates were averaged for each examined day within the 10‐year period for statistical analysis. Chi‐squared tests were used for the statistical analysis, and a *p* value <.0125 was regarded as significant following Bonferroni correction for multiple comparisons. Statistics were calculated using the MedCalc software (version 19).

## 3 RESULTS

### 3.1 Thrombolysis

Thrombolysis data are presented in Table 1.

**TABLE 1:** The total number of patients receiving thrombolytic treatment between 1 January 2007 and 31 December 2016 in Hungary in the analyzed periods before and after 24 December

Period	Dates	Women <65 years	Men <65 years	Women >65 years	Men >65 years	All patients
2 days	22/12−23/12	5	18^#^	17^#^	15	55
	25/12−26/12	3	12	11	11	37*
	Total	8	30	28	26	92
5 days	19/12−23/12	14	49^#^	40^#^	48	151
	25/12−29/12	6	30	23	16	75*
	Total	20	79	63	64	226
1 month	23/11−23/12	190	370^#^	406^#^	416	1,382
	25/12−23/01	141	311	415	353	1,220*
	Total	331	681	821	769	2,602

* *p* <.0125 versus “before” period.

^#^ *p* <.0125 versus women <65 years in the “before” period.

#### 3.1.1 Comparison of the data of the 2‐day periods preceding and following 24 december

The higher number of thrombolysis was performed in the 2‐day period before compared to that after 24 December (*χ* ^2^ = 7.001; *p* =.0081). This difference was not present when patient subgroups were analyzed.

When data preceding Christmas among patients below 65 years of age were analyzed, the proportion of men was significantly higher compared to women (*χ* ^2^ = 14.375; *p* =.0001). In the same period among women, the proportion of those over 65 years of age was significantly higher compared to those below (*χ* ^2^ = 12.791; *p* =.0003).

#### 3.1.2 Comparison of the data of the 5‐day periods preceding and following 24 december

The higher number of thrombolysis was performed in the 5‐day period before compared to that after 24 December (*χ* ^2^ = 50.977; *p* <.0001). A higher number of patients were recorded in both the subgroups below (*χ* ^2^ = 14.661; *p* =.0001) and over 65 years of age (*χ* ^2^ = 37.657; *p* <.0001). The difference is significant in the subgroups of male (*χ* ^2^ = 36.242; *p* <.0001) and female patients (*χ* ^2^ = 14.969; *p* =.0001) as well.

When data preceding Christmas among patients below 65 years of age were analyzed, the proportion of men was significantly higher compared to women (*χ* ^2^ = 38.586; *p* <.0001). In the same period among women, the proportion of those over 65 years of age was significantly higher compared to those below (*χ* ^2^ = 20.881; *p* <.0001).

#### 3.1.3 Comparison of the data of the 1‐month periods preceding and following 24 december

The higher number of thrombolysis was performed in the 1‐month period before compared to that after 24 December (*χ* ^2^ = 20.130; *p* <.0001). A higher number of patients were recorded in the subgroup below 65 years of age (*χ* ^2^ = 23.075; *p* <.0001). The difference is significant in the subgroup of male patients as well (*χ* ^2^ = 20.553; *p* <.0001).

When data preceding Christmas among patients below 65 years of age were analyzed, the proportion of men was significantly higher compared to women (*χ* ^2^ = 115.590; *p* <.0001). In the same period, the proportion of those over 65 years of age was significantly higher compared to those below among women (*χ* ^2^ = 156.419; *p* <.0001).

### 3.2 Mortality

Mortality data are presented in Table 2.

**TABLE 2:** The total number of stroke‐related deaths between 1 January 2007 and 31 December 2016 in Hungary in the analyzed periods before and after 24 December

Period	Dates	Women <65 years	Men <65 years	Women >65 years	Men >65 years	All patients
2 days	22/12–23/12	41	93^#^	505^#^	289^&^, ^$^	928
	25/12–26/12	40	87	523	311	961
	Total	81	180	1,028	600	1,889
5 days	19/12–23/12	122	224^#^	1,242^#^	736^&^, ^$^	2,224
	25/12–29/12	100	220	1,133*	772	2,325
	Total	222	444	2,375	1,508	4,549
1 month	23/11–23/12	629	1,300^#^	6,710^#^	4,249^&^, ^$^	12,888
	25/12–23/01	631	1,288*	6,837	4,440	13,196*
	Total	1,260	2,588	13,547	8,689	26,084

* *p* < .0125 versus “before” period.

^#^ *p* < .0125 versus women <65 years in the “before” period.

^&^ *p* < .0125 versus women >65 years in the “before” period.

^$^ *p* < .0125 versus men <65 years in the “before” period.

#### 3.2.1 Comparison of the data of the 2‐day periods preceding and following 24 december

No significant difference could be detected in mortality between the compared periods in terms of the whole sample or within the subgroups (based on sex and age).

When data preceding Christmas were analyzed, a higher number of deaths were recorded in case of men compared to women below 65 years of age (*χ* ^2^ = 40.195; *p* <.0001), whereas over 65 years of age, a significantly higher number of deaths were recorded in case of women compared to men (*χ* ^2^ = 117.413; *p* <.0001). Higher numbers of deaths were recorded in the age group over compared to below 65 years of age in both female (*χ* ^2^ = 788.248; *p* <.0001) and male patients (*χ* ^2^ = 200.798; *p* <.0001).

#### 3.2.2 Comparison of the data of the 5‐day periods preceding and following 24 december

No significant difference could be detected in mortality between the compared periods in the whole sample. However, in case of women over (*χ* ^2^ = 7.598; *p* =.0058) 65 years of age, a higher number of deaths were recorded during the 5‐day period before 24 December compared to that after the feast.

When data preceding Christmas were analyzed, a higher number of deaths were recorded in men compared to women below 65 years of age (*χ* ^2^ = 60.053; *p* <.0001), whereas in the age group over 65 years of age, a higher number of deaths were recorded in women compared to men (*χ* ^2^ = 175.193; *p* <.0001). In case of both women (*χ* ^2^ = 706.248; *p* <.0001) and men (*χ* ^2^ = 200.798; *p* <.0001), a higher number of deaths were recorded in the age group over 65 years compared to below 65 years of age.

#### 3.2.3 Comparison of the data of the 1‐month periods preceding and following 24 december

In the whole sample, a higher number of deaths were recorded during the 1‐month period after compared to that before 24 December (*χ* ^2^ = 7.512; *p* =.0061). As for the subgroup analysis, only in the subgroup of men below 65 years of age was the number of deaths higher before compared to after 24 December (*χ* ^2^ = 8.410; *p* =.0037).

When data preceding Christmas were analyzed, a higher number of deaths were recorded in men compared to women below 65 years of age (*χ* ^2^ = 466.561; *p* <.0001), whereas in the age group over 65 years of age, a higher number of deaths were recorded in women compared to men (*χ* ^2^ = 1,105.607; *p* <.0001). In case of both women (*χ* ^2^ = 10,076.905; *p* <.0001) and men (*χ* ^2^ = 3,133.637; *p* <.0001), a higher number of deaths were recorded in the age group over 65 years compared to below 65 years of age.

## 4 DISCUSSION

Identifying associations between cardiovascular events such as the onset of stroke and various events may provide potential benefit in terms of prevention. Accumulation of cardiovascular events including stroke during winter, early spring, and cold weather is well documented (Azevedo et al., 1995; Tsementzis et al., 1991). This can be well explained by biological reasons (i.e., sympathicotonia) and the effects of certain meteorological factors (Jiminez‐Conde et al., 2008). Among the days of the week, Monday is a risk factor, whereas the weekend can be regarded as a period with reduced risk (Folyovich et al., 2018). A number of biases can hamper the identification of stroke onset; for example, many patients do not seek medical help on their days off. In addition, psychological factors can play significant roles, such as the increased level of anxiety associated with returning to work (Folyovich et al., 2018; Manfredini et al., 2009). The accumulation of stroke events in the end of the month, however, must be merely associated with psychological causes, as there is no biological factor that would occur exclusively on the last day of the month (Folyovich et al., 2016). It is yet to be determined whether other periods that are associated with significant emotional impact could have an influence on cardiovascular events. Feasts play a significant role for both the society and the individual, out of which Christmas is of utmost significance in Hungary.

The unfavorable impacts of psychological factors on cardiovascular events have been studied before by a number of groups. Depression is an independent risk factor of myocardial infarction, and its role has been studied in stroke as well (Pan et al., 2011). Psychosocial stress has a negative influence on circulation disorders (Kopp & Réthelyi, 2004). The incidence of myocardial infarction recurrently emerges in the periods of the year that are more stressful for the population. The hypothesis of stress‐evoked infarction was studied in a Swedish population based on the symptom onset and the time of hospital admission (Wallert et al., 2017). The authors analyzed the period of Christmas as well. This was defined as a 3‐week period (from 15 December till 6 January) and was found to be associated with a higher incidence of myocardial infarction. This risk, similarly to our present findings, was predominantly true for pensioners. A possible explanation for this phenomenon is that active employees are younger and healthier, and the stress experienced during the holidays may prevail among the particularly sensitive pensioners. Active employees experience both the stress‐increasing effect of winter holiday and the stress‐decreasing effect of recreation.

Another study examining people living in distant cultures found that the mortality decreases before and during feasts of emotional importance, and increases thereafter (Phillips & King, 1988; Phillips & Smith, 1990). In other words, the death “shifts.” Mortality has been studied in association with the “Harvest Moon Festival,” an autumn feast important for the Chinese (and other Asian nationalities). The “shift” of mortality to the period after the feast was clearly observable. This festivity is not celebrated on the same day each year; therefore, it is especially suitable for the analysis of psychological impacts. It is interesting that stroke more sensitively showed this phenomenon than myocardial infarction. The mortality of elderly Chinese women reflected this effect the most pronouncedly, who are the very central participants of the ceremonies. The shift of mortality to a period that is after the religious feast can also be observed in the Jewish population. During Pesach (or Passover), this phenomenon can be observed most markedly in Jewish men, who, on the other hand, are the key figures of their feast. The intense emotional impact of feasts is further evidenced by the study of Jessen and Jensen, who analyzed the incidence of suicide in 32.291 Danish people over 15 years of age in a 25‐year period (Jessen & Jensen, 1999). This study provided evidence for the “broken promise effect”: significantly more suicide attempts occurred (i.e., “postponed” until) after the feast than before. This phenomenon was most pronounced around Christmas, Easter, and Pentecost.

According to the findings of an American study analyzing data of a 25‐year period, both Christmas and New Year's Eve are risk factors for mortality (Phillips et al., 2010). The increase in mortality was observable in a number of disease groups, including circulation disorders, tumors, respiratory diseases, and endocrine and metabolic disorders. The study of Knight et al. (Knight et al., 2016) provided a particularly valuable aspect in terms of the evaluation of the impact of Christmas, as they analyzed data from New Zealand, where Christmas is in the summer. This season is usually associated with lower cardiovascular and stroke mortality; therefore, the transient increase observed by this study cannot be linked to anything else but the feast itself. The authors confirmed by analyzing a period of 25 years that in the interval between 25 December and 7 January, the (out‐of‐hospital) cardiac mortality increased with 4.2 percent. They also observed that the average age of individuals deceased in this period was almost 1 year lower than of those who died in other periods of the year. Lapointe‐Shaw et al. reported that in patients discharged from hospital for the December holidays (i.e., Christmas and New Year's Eve), the risk of mortality and readmission to emergency departments were higher. The period of highest risk was the week before and after Christmas, and the risk was highest within 7 days after discharge (Lapointe‐Shaw et al., 2018).

Several explanations have been proposed for the role of Christmas as a cardiovascular risk factor (Phillips et al., 2004, 2010): (a) People are exposed to significant psychological stress during the holidays; (b) they are usually affected by a positive emotional impact, which is suggested to be in association with the phenomenon of “shifted mortality” (these phenomena can be observed to a less extent in Alzheimer's disease patients); (c) traveling is more frequent during holidays and days off, which has its own risk factors and thus a higher rate of mortality; d) less people seek health care during holidays; (e) cold weather is a risk factor of cardiovascular diseases due to its sympathicotonic effect; (f) periods associated with cold weather also have an increased incidence of respiratory diseases, including flu and pneumonia, which are likewise cardiovascular risk factors; and (g) holidays are often associated with pathological amount of food consumption, as well as significant alcohol intake and illicit drug use.

The novelty of our study is that it not only analyzed the number of deaths in relation to an important feast, but it also examined the number of thrombolysis in this respect, a reliable marker that is suitable for the estimation of acute stroke frequency. The narrow time window and precise documentation of thrombolytic interventions enable the precise identification of the onset of stroke, narrowed to a 1–5‐hr interval. A limitation of our study is that the religious affiliation of patients is not known during hospital care. This, nevertheless, is not in contradiction with choosing Christmas as the subject of our investigation. Disclosure of religious affiliation is not mandatory in Hungary. However, Christian churches are predominantly represented (approximately 72.4% of the population). Nevertheless, many of the traditionally non‐Christian believers (in many cases, Jewish people living in interfaith marriage) and atheists do celebrate Christmas as well; therefore, we consider this feast to be a reliable indicator at the society level as well.

Previous reports choosing mortality as an indicator established the death “shift” theory (Phillips & King, 1988; Phillips & Smith, 1990), which means that the death of persons particularly affected emotionally by a given social–religious event or ceremony is postponed until after the feast. While overall stroke‐associated mortality was indeed increased 1 month after Christmas in our present study, the death rate was higher a month before rather than after Christmas in men below 65 years of age and in women over 65 years of age 5 days before Christmas. Furthermore, increased stroke incidence also accumulated up to 1 month before Christmas, particularly in men below 65 years of age. On the other hand, in the population over 65 years of age, women were clearly more affected than men, probably due to their prominent role (increased household tasks and emotional devotion) in the feast. Overall, based on the above, the previously described death “shift” phenomenon could not be demonstrated in this study. On the contrary, we suggest that in case of elderly women, a subgroup that is otherwise somatically vulnerable, this minimal emotional excess is sufficient to provoke the disease, most prominently in the 5 days preceding Christmas, while in men below 65 years, a group probably less affected by Christmas‐related preparations, the increased psychological stress of providing financial security for the celebration may play a role up to 1 month before Christmas. Therefore, from a preventative perspective, public health messaging in the media at least a month in advance before the feast should warn this at‐risk group in a targeted manner to empower them to deal with this increased stress.

In conclusion, this study provides evidence that the number of thrombolytic treatments for acute ischemic stroke is significantly higher in the periods (2 days, 5 days, and 1 month) preceding Christmas in Hungary compared to the corresponding period that follows the feast. This increase is particularly prominent in men below 65 years of age. Stroke‐related mortality is also increased in two subgroups before Christmas: in men below 65 years of age 1 month before the feast and in women above 65 years of age 5 days before the feast. These findings may predominantly relate to emotional and psychological factors. The anxiety secondary to festive preparations and “mandatory” spending and gift purchasing can be suggested as contributors. Unveiling the exact cause and the validation of our results in other populations, however, requires further investigations.

## CONFLICT OF INTEREST

The authors have no potential conflicts of interest to disclose.

## AUTHOR CONTRIBUTION

AF and TJ designed study. DJ and MP curated data. RM and NAM performed statistical analysis. AF and AKBM wrote manuscript. ET and GT revised manuscript.

## Data Availability

The data that support the findings of this study are available from the corresponding author upon reasonable request.
